# Extended approach to sum of absolute differences method for improved identification of periods in biomedical time series

**DOI:** 10.1016/j.mex.2020.101094

**Published:** 2020-10-09

**Authors:** Tomasz Wiktorski, Aleksandra Królak

**Affiliations:** a Department of Electrical Engineering and Computer Science, University of Stavanger, Norway; b Institute of Electronics, Lodz University of Technology, Poland

**Keywords:** Moving average, SAD, Heart rate, Third gradient, Uncertainty factor

## Abstract

Time series are a common data type in biomedical applications. Examples include heart rate, power output, and ECG. One of the typical analysis methods is to determine longest period a subject spent over a given heart rate threshold. While it might seem simple to find and measure such periods, biomedical data are often subject to significant noise and physiological artifacts. As a result, simple threshold calculations might not provide correct or expected results. A common way to improve such calculations is to use moving average filter. Length of the window is often determined using sum of absolute differences for various windows sizes. However, for real life biomedical data such approach might lead to extremely long windows that undesirably remove physiological information from the data. In this paper, we:

• propose a new approach to finding windows length using zero-points of third gradient (jerk) of Sum of Absolute Differences method;
• demonstrate how these points can be used to determine periods and area over a given threshold with and without uncertainty.

We demonstrate validity of this approach on the PAMAP2 Physical Activity Monitoring Data Set, an open dataset from the UCI Machine Learning Repository, as well as on the PhysioNet Simultaneous Physiological Measurements dataset. It shows that first zero-point usually falls at around 8 and 5 second window length respectively, while second zero-point usually falls between 16 and 24 and 8–16 s respectively. The value for the first zero-point can remove simple measurement errors when data are recorded once every few seconds. The value for the second zero-point corresponds well with what is known about physiological response of heart to changing load.

## Graphical abstract

**Figure:**
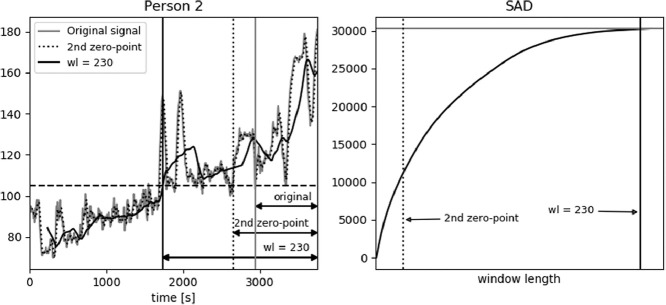

Specifications Table

**Table:** 

Subject Area:	Computer Science
More specific subject area:	Biomedical, time series data analysis
Method name:	Sum of Absolute Differences
Name and reference of original method:	J. Moorer, "The optimum comb method of pitch period analysis of continuous digitized speech," in IEEE Transactions on Acoustics, Speech, and Signal Processing, vol. 22, no. 5, pp. 330–338, October 1974.
Resource availability:	https://archive.ics.uci.edu/ml/datasets/PAMAP2+Physical+Activity+Monitoringht https://physionet.org/content/simultaneous-measurements/1.0.0/

## Method details

Signals from sensors usually contain some amount of noise that can have a negative impact on further data analysis. Usually, such signals are pre-processed with a form of a low-pass filter to remove unwanted elements. One of the typical and simplest low-pass filters is a moving average filter. In this filter a window of a certain length is continuously applied through the signal and value for the current time step is substituted for average of current and adjacent steps. The length of the window is decided either using domain's rule of thumb or by using Sum of Absolute Differences (SAD) approach. However, SAD approach might lead to too large window length for some biomedical signals, e.g. heart rate.

In the regular SAD approach, a point is identified at which the SAD curve flattens. This point is then selected as the window length. We propose an extension to the SAD approach with use of third gradient, also called jerk, of the SAD curve. Zero-points of the third gradient become suggested window lengths. First point would be considered a conservative value, which preserves most information, but might also not remove all the noise. While second zero-point would be considered a liberal value, which guarantees that most noise is removed while still preserving most important information.

### General approach to sum of absolute differences

In SAD approach, a set of filtered versions of the signal is calculated for varying window lengths. Then each of filtered signals is subtracted from the original one, point by point. Absolute values of each point difference are then summed. This results in set of sums, each for different window length. These sums when plotted form a curve that usually flattens from a certain window length, suggesting that there is no further filtering effect for longer windows. This window length is then selected as the optimal for filtering the signal for further analysis.

**Fig. 1:**
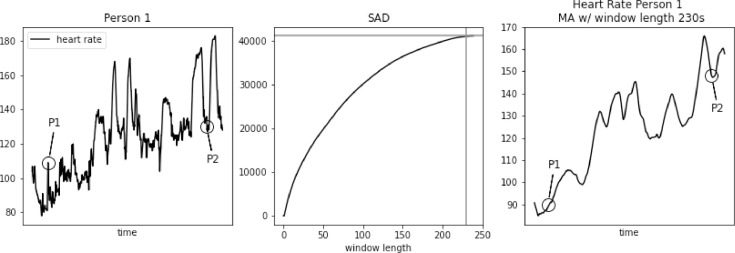
Heart rate, sum of absolute differences, and heart rate with moving average filter with window length based on usual SAD approach, for Person 1.

**Fig. 2:**
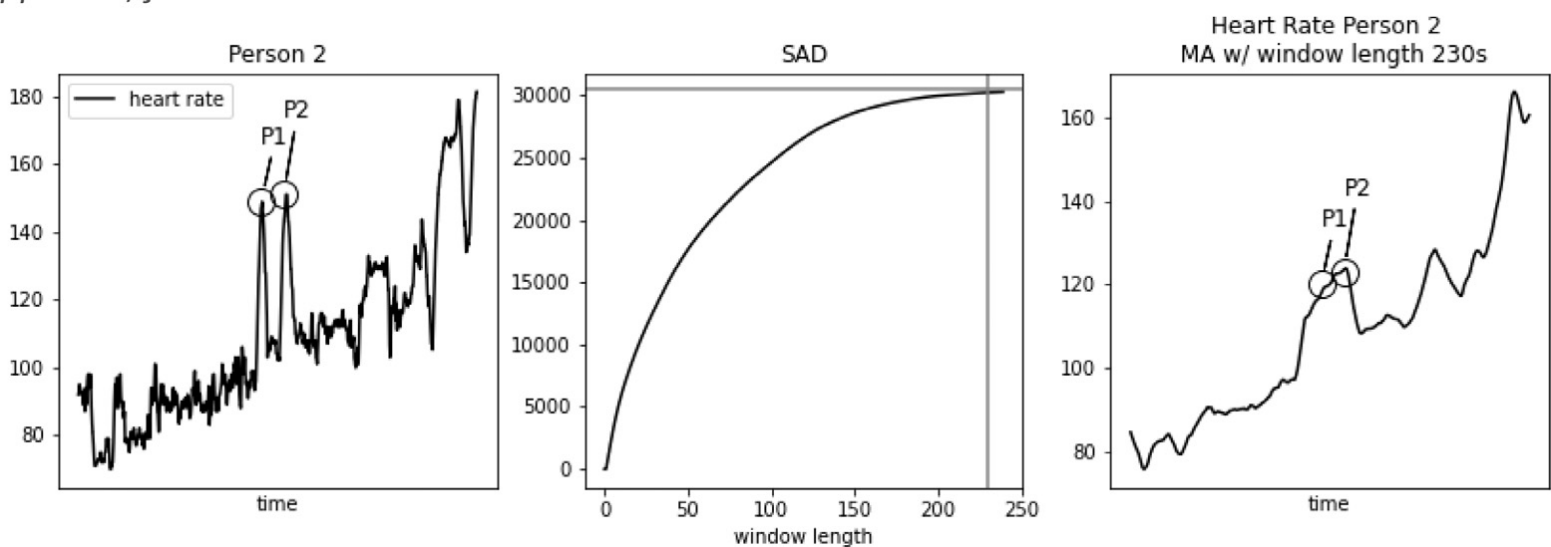
Heart rate, sum of absolute differences, and heart rate with moving average filter with window length based on usual SAD approach, for Person 2.

**Fig. 3:**
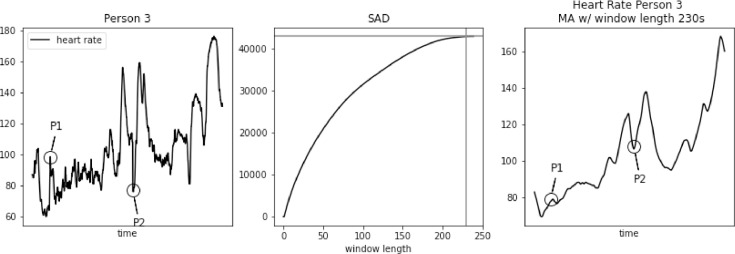
Heart rate, sum of absolute differences, and heart rate with moving average filter with window length based on usual SAD approach, for Person 3.

**Fig. 4:**
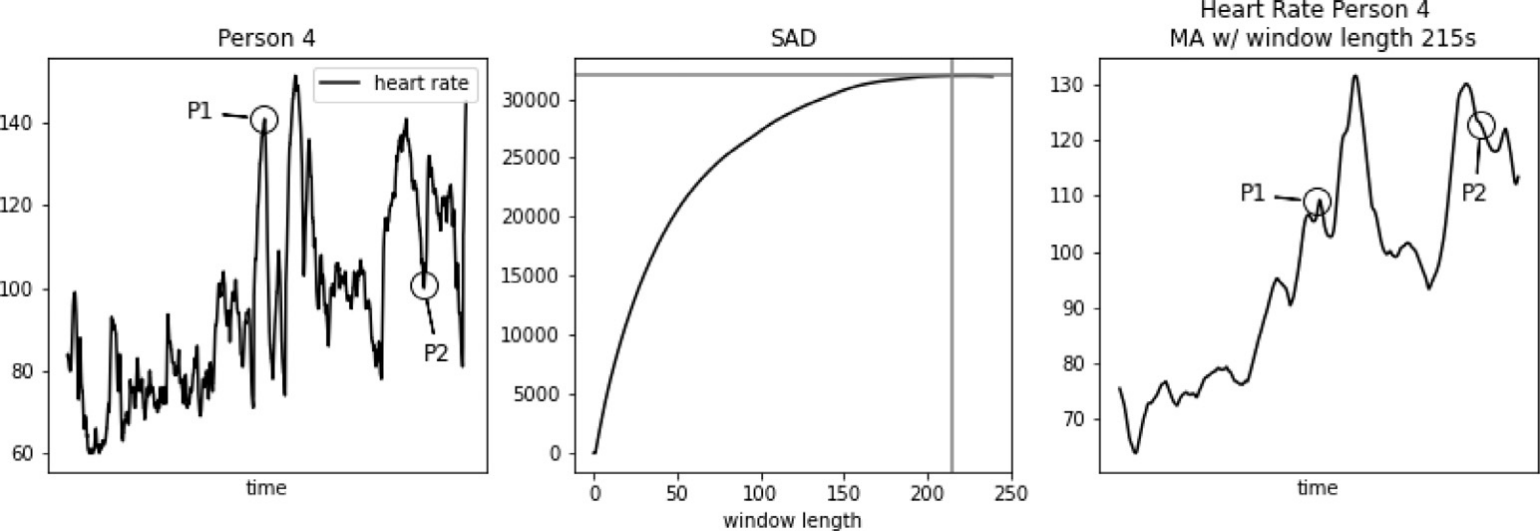
Heart rate, sum of absolute differences, and heart rate with moving average filter with window length based on usual SAD approach, for Person 4.

**Fig. 5:**
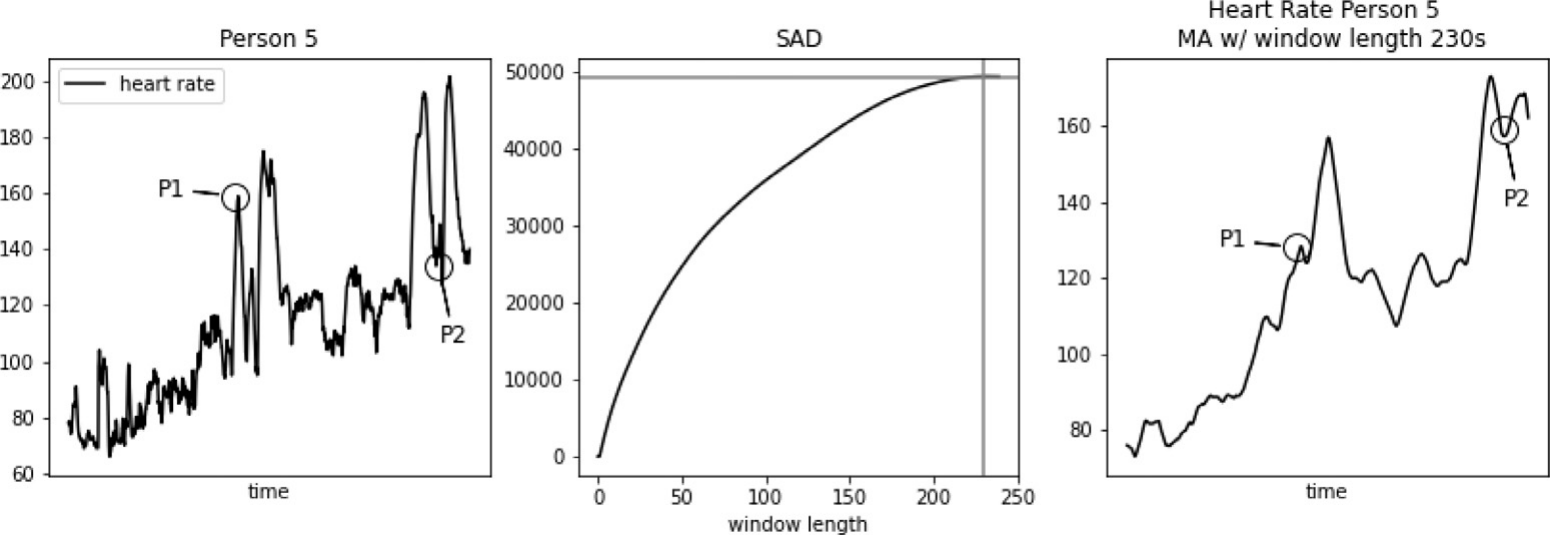
Heart rate, sum of absolute differences, and heart rate with moving average filter with window length based on usual SAD approach, for Person 5.

However, SAD approach might lead to too large window length for some biomedical signals, e.g. heart rate. As we demonstrate in Fig. 1, Fig. 2, Fig. 3, Fig. 4, Fig. 5 using PAMAP2 [5] dataset and in Fig. 12, Fig. 13, Fig. 14 using “Simultaneous physiological measurements with five devices at different cognitive and physical loads” dataset from PhysioNet database [11,12] the resulting window length would become 210–230 or 490–590 s respectively for each dataset using the established approach. It is not only intuitively too long, but also would mask physiological response to a change in physical exertion that becomes visible in less than a minute [9]. Beyond that point a change can still be observed, but it is then related to cardiac drift when maintaining a level of increased exertion [4]. In such a case, selecting window length based on the SAD approach would result in significant loss of information in the available data.

The loss of information is clearly visible in the examples. In Fig. 1a significant increase in heart rate at point P1 from the original dataset completely disappears after applying Moving Average (MA) filter with window length 230 s, obtained using regular SAD approach. A drop in heart rate value at point P2 is represented, but its lowest value is around 25 bpm higher than in the original dataset. Such difference is physiologically significant.

In Fig. 2 we focus on two significant spikes in heart rate at points P1 and P2. Their value is around 40 bpm higher than the surrounding values. However, after applying MA filter with window length 230 s, the curve tells a completely different story. Points P1 and P2 are part of a consistent heart rate increase. As a result, filtering might lead to a complete change in the interpretation of these data.

In Fig. 3, Fig. 4, Fig. 5 we further observe how the wrong choice of window length alters characteristic points of the signal, to an extent that these points blend with the remaining signal. It happens both for spikes and drops. For point P1 in each of these three figures we notice that a strong spike in heart rate is greatly reduced. While still visible, it hardly stands out of the general trend. For point P2 in each of these three figures a significant drop is reduced to a minor one. Reduction in relative value, to the spikes that often surround such drops, ranges between 50 and 30 bpm.

While it might seem that a general shape of the heart rate curve is preserved after applying MA filter with a window length based on regular SAD approach, we observe that many significant details are lost. This observation is consistent with typical physiological response of heart rate that should be visible in less than a minute [4]. What suggests, therefore, that a window length for a MA filter should not be longer.

### Zero-Points of third gradient (Jerk)

If we consider continuous function *g(x)*, its derivative can be defined as its rate of change with respect to variable *x*. If function *g(x)* is a function of one variable, its derivative is the same as partial derivative, that can be calculated as a dot product of the gradient of the function *g(x)* and unit vector *u* [1]. In case of uniformly distributed time series with step equal to 1, we can assume that the unit vector *u* is equal to 1. It leads us to conclusion that in case of discrete function *g(i)* with step *h = 1*, its derivative is equal to the gradient of this function.

The first gradient of the discrete function *g(i)* is calculated as presented in Eg. (1) [2]:

(1) $$\hat{g}{\left(i\right)}^{\left(1\right)}=\frac{g\left(i+1\right)-g\left(i-1\right)}{2\cdot h}$$

The calculated gradient may be burdened with an error defined by the limiting behavior of a step function *h*, denoted by *O(h^2^)*. In case of regular sampling of the discrete time series *g(i)* with *h* = 1 the gradient of *g(i)* can be calculated as shown in Eq. (2):

(2) $$\hat{g}{\left(i\right)}^{\left(1\right)}=0.5\left(g\left(i+1\right)-g\left(i-1\right)\right)$$

We can detect finer changes in the behavior of SAD curve using zero-points of the third gradient of the curve. The third gradient of a discrete function *g(i)* can be considered as the third derivative of *g(i)*, should *g(i)* be a continuous function. It can be understood more intuitively by comparison to the third derivative of position in physics, which is known as jerk, the time derivative of acceleration [3]. For example, the effect of acceleration is the feeling of being pressed into the seat of a car. If the acceleration is not constant, we can talk about jerk that is felt as an increasing or decreasing force on the body.

Calculation of zero crossing point of the third gradient of function *g(i)* can be expressed by Eq. (3):

(3) $$i_{z-c}=i\;if\;\hat{g}{\left(i-1\right)}^{\left(3\right)}>0\;and\;\hat{g}{\left(i\right)}^{\left(3\right)}<0\;or\;\hat{g}{\left(i-1\right)}^{\left(3\right)}\langle 0\;and\;\hat{g}{\left(i\right)}^{\left(3\right)}\rangle 0$$

If the subsequent zero-points are located less than 3 time-steps apart, then we suggest combining them with the resulting value calculated as a ceiling of an arithmetic mean of these zero-points (Eq. (4)).

(4) $$i_{z-c}=\left\lceil \;\left(\bar{i_{z-c1},i_{z-c2}}\;\right)\right\rceil$$

In Algorithm 1 we present an outline of a simple algorithm to calculate first three zero-points of third gradient of SAD curve for a given signal *TS*. First, in addition to the input signal, we specify *N* as a maximum length of SAD curve. In line 1 we create an empty list that will later hold values of sum of absolute differences for different window length of a moving average filter. These values are calculated in lines 2 and 3.

**Algorithm 1:** Algorithm for calculating first three zero-points of SAD curve for a given signal.

**Data**: TS := [v_1_,…v_t_,…,v_T_]*list of T time-ordered values* N := 250*maximum length of SAD curve* **Result**: ZP := [P_1_, P_2_, P_3_]*list of 3 zero-points* 1 SAD_curve := []*empty list for values of sum of absolute differences* 2 for n in N: 3 SAD_curve.append((n, sum(abs(TS-moving_avg(TS,n)))) 4 3_gradient := gradient(gradient(gradient(SAD_curve))) 5 ZP := where_diff(sign(3_gradient))[:3]

In line 4 we calculate third gradient of the SAD curve. Definition of gradient function is provided earlier in this section and implementation of such function is available in major data analytics tools, e.g. in NumPy. Finally, in line 5, we extract only the sign of third gradient values and check for change in this sign. We select first three such places in the curve. The algorithm can be further extended by combining zero-points located closely to each other.

### Selecting moving average window size and calculating typical measures

Later, in method validation, we demonstrate that the first zero-point of jerk corresponds to 5–8 s long window for moving average filter. It is therefore safe to assume that any signal variation in that range would be related to simple measurement errors when data are recorded once every few seconds. Second zero-point of jerk corresponds to 16–24 s window for moving average. In that range physiological effect should already be visible [9].

As a result, there are at least two values that could be applied in further processing. We suggest two possible approaches to how these values can be used. First approach uses one of these two zero-points of jerk as the window size for a moving average filter and any further calculations are performed in a standard way on the filtered signal. Second approach is approximate and combines the two values together with an uncertainty factor inspired by the interval type-2 fuzzy set footprint of uncertainty [10].

Two of the most typical cumulative measures used to evaluate the condition of a person based on the heart rate trace are: (1) longest period above threshold value of HR, and (2) area under the plot for HR greater than the threshold value. We present how these measures are formally calculated, before using them for validation.

In order to calculate the longest period above the threshold value *thr* of HR we define *S* to be the set of periods *s_m_* for the time series *HR*, where each *s_m_* is a series of subsequent values from *HR* having values *HR*(*i*) ≥ *thr* (Eq. (5)). Total number of periods *s_m_* in given *HR* series is assumed to be equal to *M*.

(5) $$S={\left\{s_{m}\right\}}_{m=1}^{M}$$

Single element *s_m_* of set *S* has length *l_m_* that is defined by Eq. (6.):

(6) $$l_{m}=i_{stop}-i_{start}\;\text{where}\;\left\{ \begin{array}{c} i_{start}=i\;such\;that\;HR\left(i\right)\geq thr\;and\;HR\left(i-1\right)<thr\\ i_{stop}=i\;such\;that\;HR\left(i\right)\geq thr\;and\;HR\left(i+1\right)<thr\\ \forall_{i\epsilon \left(i_{start},i_{stop}\right)}\;\;HR\left(i\right)\geq thr\;\\ i_{stop}>i_{start}\\ i\in \left[0,I\right] \end{array}\right.$$

We define *L* as the set of lengths *l_m_*. The longest period above the threshold value is denoted as *l_max_* and is the maximum value of a set *L* (Eq. (7)):

(7) $$l_{max}=\max L$$

Energy is considered as the area *A* under the plot above the threshold value and is calculated as shown in Eq. (8):

(8) $$A=\left(\sum_{i=0}^{I}HR{\left(i\right)}_{\geq thr}\right)-n\cdot thr$$

Number of elements in the *HR* sequence is defined as *I* and *n* is the number of samples in sequence *HR* greater or equal to *thr* value. Area under the plot can be expressed in beats: *heart* *rate*[*bpm*]*time[*min*], or *heart* *rate*[*bpm*]*time[*sec*]/60. In case of the area under the plot and above the threshold value, it is equal to the number of total beats over the threshold.

**Fig. 6:**
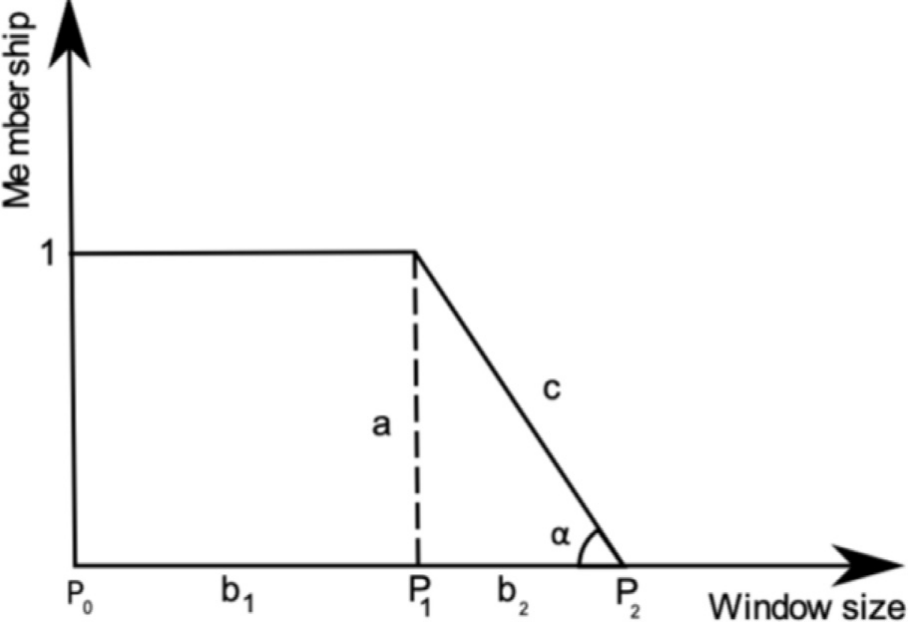
Type-2 fuzzy set membership function with footprint of uncertainty applied to two zero-points of jerk.

In Fig. 6 we present a typical plot of type-2 fuzzy set membership function with footprint of uncertainty applied to two zero-points of third gradient of SAD curve.

We define length adjusted by the uncertainty factor as given in Eq. (9):

(9) $$l_{adj}=l_{2}\cdot UF+l_{1}$$

where:

*l_1_* – length of the longest segment above the threshold for sampling time equal to 1st zero-point of jerk
*l_2_* – length of the longest segment above the threshold for sampling time equal to 2nd zero-point of jerk

We define uncertainty factor in Eqs. (10) and 11:

(10) $$UF=\sin\alpha =\frac{a}{c}$$

(11) $$c=\sqrt{b_{2}^{2}+1}$$

where:

a = 1
b_1_ – distance between 0 and point P_1_
b_2_ – distance between points P_1_ and P_2_
P_1_ and P_2_ – window sizes corresponding to 1st and 2nd zero-point of jerk

For example, for *P_1_*=5, *P_2_*=22, *l_1_=950*, and *l_2_=1298;* we have *b_1_*=5 and *b_2_*=17 and consequently:

$$c=\sqrt{17^{2}+1}=17.03$$

$$UF=\sin\alpha =\frac{1}{c}=0.06$$

$$l_{adj}=0.06\cdot 1298+950=1028$$

We define area adjusted by the uncertainty factor in Eq. (12):

(12) $$A_{adj}=\left(A_{2}-A_{1}\right)\cdot UF+A_{1}$$

where:

*A_1_* – area under the HR curve above the threshold for Moving Average filter with window length equal to 1st zero-point of jerk.
*A_2_* – area under the HR curve above the threshold for Moving Average filter with window length equal to 2nd zero-point of jerk.

Using values from the example with *A_1_= 66,253*, and *A_2_= 57,314* we obtain:

$$A_{adj}=\left(,573,14-,662,53\right)\cdot 0.06+66,253=65,728$$

## Method validation

We demonstrate the validity of the proposed method in three different ways. First, we show that most important characteristics of the heart rate curve are preserved, when using first or second zero-point of third gradient of SAD as the window length for a MA filter. We also make a connection between values of the zero-points and physiological phenomena related to heart rate development. Subsequently, we demonstrate improved results in calculating longest period over a given threshold value, based on the filtered data. Finally, we discuss an impact of MA filtering on integral of heart rate over a given threshold value. It is another common statistic, that might be impacted in a non-obvious way by filtering.

### Part 1 of validation – shape of HR curve

We observed earlier, in Fig. 1, Fig. 2, Fig. 3, Fig. 4, Fig. 5, that applying MA filter with window length obtained using regular SAD approach leads to loss of significant details in heart rate signal. Moreover, such window length appears also to conceal physiological phenomena.

We applied the modified SAD approach using zero-points of third gradient, as defined in the previous section, to the same PAMAP2 data. First three zero-points were calculated. For the first zero-point values ranged from 5 to 8 s for different persons. For the second zero-point values ranged from 16 to 24 s. For the third zero-point values ranged from 21 to 32 s.

It is interesting to notice that all these zero-points lie with the physiological response range for the heart rate [4]. First zero-point is at the very beginning of that range, so it can ensure that all physiological phenomena will still be visible in the filtered data. Window with the length corresponding to the second zero-point offers more smoothing, since it is almost triple the value, but it still fits well within the physiological range. Using the third zero-point might still be useful, but in some cases the window might be considered too long. Especially, when it exceeds 30 s.

Based on these observations, first and second zero-points seem to be of most interest. With first zero-point being a safe choice. While the second zero-point is potentially optimal, balancing smoothing effect with detail preservation.

**Fig. 7:**
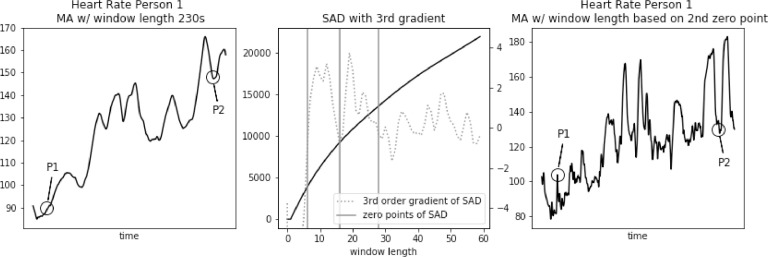
Heart rate, sum of absolute differences with third gradient, and heart rate with moving average filter with window length based on second zero-point of third gradient, for Person 1 from PAMAP2 dataset.

**Fig. 8:**
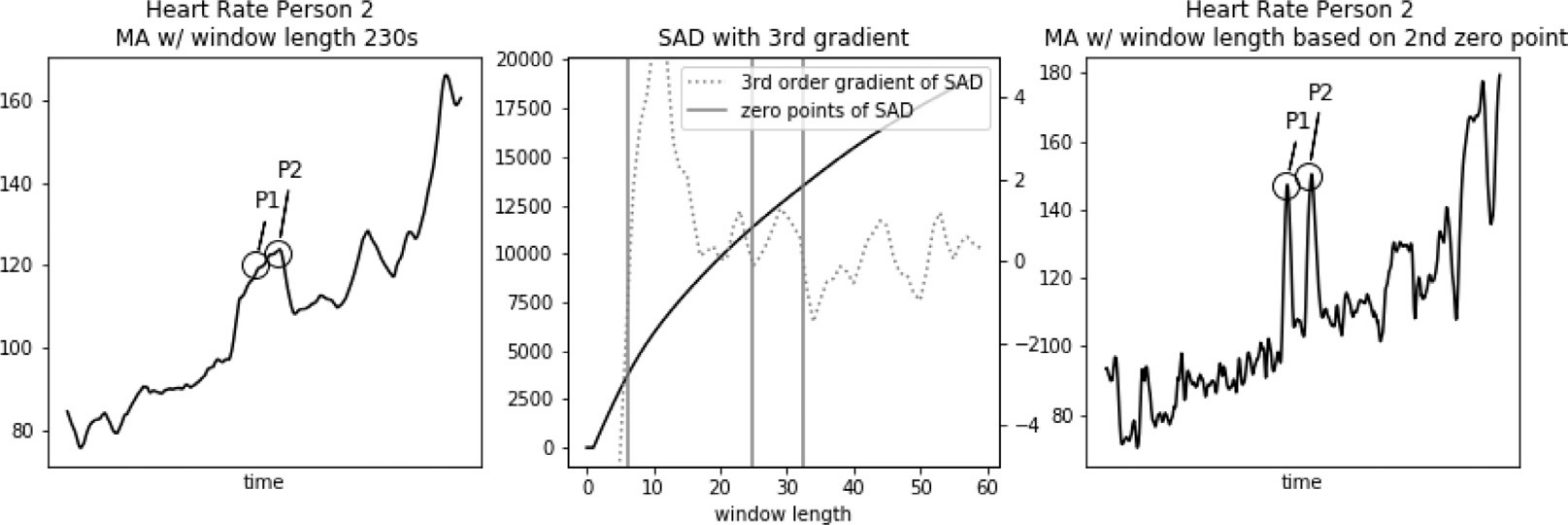
Heart rate, Sum of Absolute Differences with third gradient, and Heart Rate with Moving Average filter with window length based on second zero-point of third gradient, for Person 2 from PAMAP2 dataset.

**Fig. 9:**
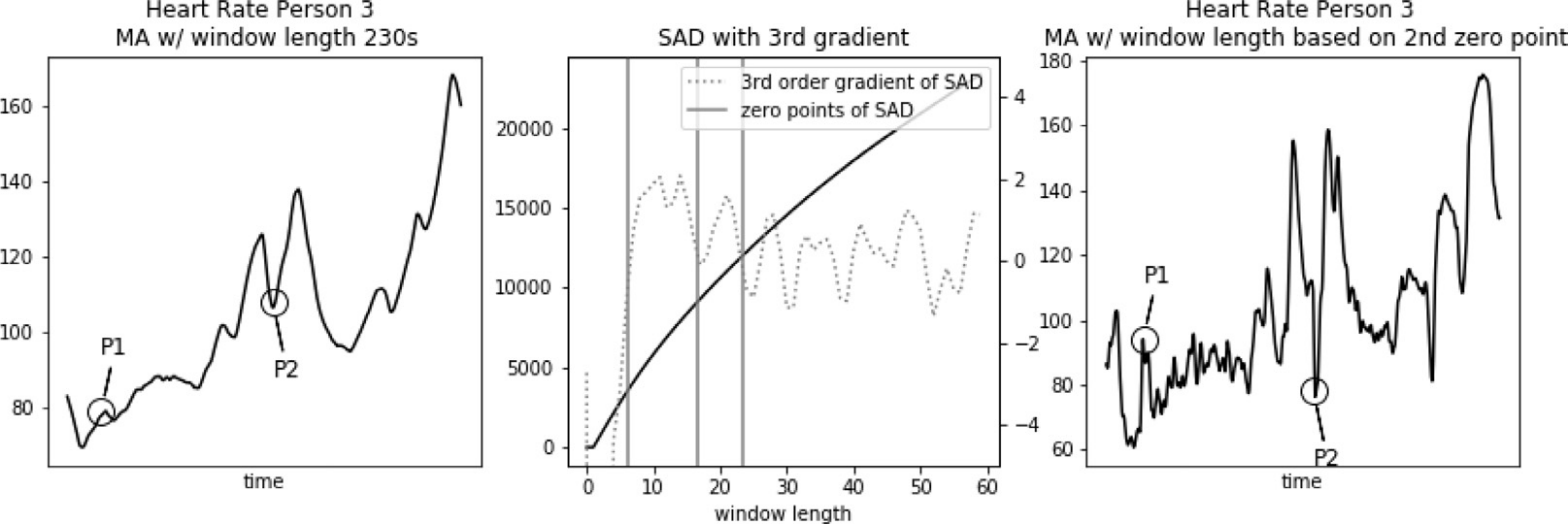
Heart rate, sum of absolute differences with third gradient, and heart rate with moving average filter with window length based on second zero-point of third gradient, for Person 3 from PAMAP2 dataset.

**Fig. 10:**
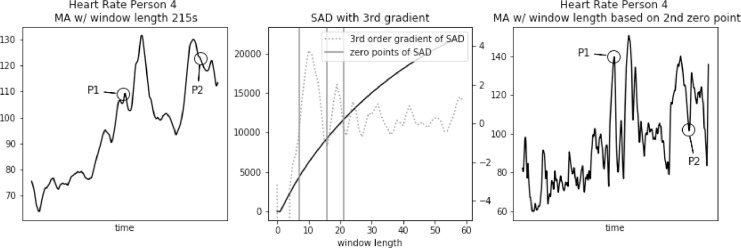
Heart rate, sum of absolute differences with third gradient, and heart rate with moving average filter with window length based on second zero-point of third gradient, for Person 4 from PAMAP2 dataset.

**Fig. 11:**
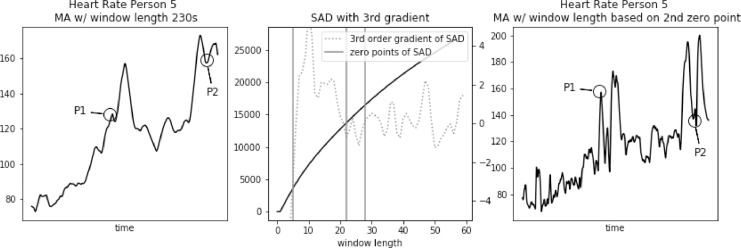
Heart rate, sum of absolute differences with third gradient, and heart rate with moving average filter with window length based on second zero-point of third gradient, for Person 5 from PAMAP2 dataset.

In Fig. 7, Fig. 8, Fig. 9, Fig. 10, Fig. 11 we apply the modified SAD approach to the same persons from PAMAP2 dataset as in Fig. 1, Fig. 2, Fig. 3, Fig. 4, Fig. 5. Data filtered with MA filter with window length selected using standard SAD approach are presented in the left part of each figure. First three zero-points of third gradient of SAD curve are calculated and presented in the middle part of each figure. Finally, the filtered signal using second zero-point is presented in the right part of each figure.

We can notice, that in each case the shape of the heart rate curve is well preserved. All characteristic points that were misrepresented using standard SAD approach, are represented correctly with the modified SAD approach.

In Fig. 7a significant increase in heart rate at point P1 from the original dataset is well represented applying Moving Average (MA) filter with window length of 8 s, obtained using modified SAD approach. A drop in heart rate value at point P2 is represented with little difference in the absolute value.

In Fig. 8 we focus on two significant spikes in heart rate at points P1 and P2. Their value is around 40 bpm higher than the surrounding values. This characteristic is preserved in the signal filtered with window length of 25 s, corresponding to the second zero-point of third gradient of the SAD curve. So, the interpretation of these data is preserved after the filtering.

In Fig. 9, Fig. 10, Fig. 11 we further observe how the window length corresponding to the second zero-point preserves characteristics of the signal. Both spikes and drops maintain the right shape. For point P1 in each of these three figures we notice that a strong spike in heart rate is preserved. For point P2 in each of these three figures significant drops are reduced only by a few bits per minute.

**Fig. 12:**
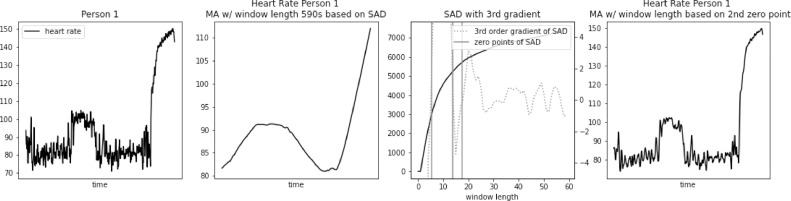
Heart rate, heart rate with moving average filter with window length based on usual SAD approach, sum of absolute differences with third gradient, and heart rate with moving average filter with window length based on second zero-point of third gradient, for Person 1 from simultaneous physiological measurements dataset.

**Fig. 13:**
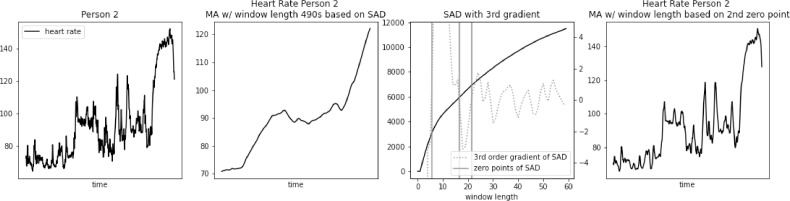
Heart rate, heart rate with moving average filter with window length based on usual SAD approach, sum of absolute differences with third gradient, and heart rate with moving average filter with window length based on second zero-point of third gradient, for Person 2 from simultaneous physiological measurements dataset.

**Fig. 14:**
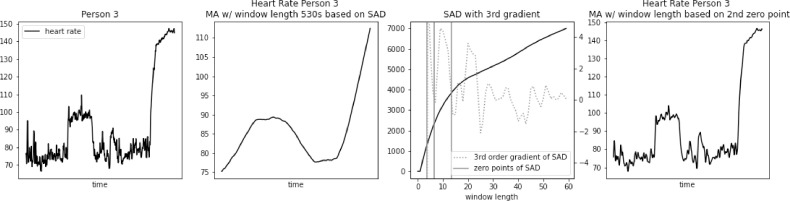
Heart rate, heart rate with moving average filter with window length based on usual SAD approach, sum of absolute differences with third gradient, and heart rate with moving average filter with window length based on second zero-point of third gradient, for Person 3 from simultaneous physiological measurements dataset.

The proposed approach was further validated using PhysioNet dataset “Simultaneous physiological measurements with five devices at different cognitive and physical loads”. Results for three example subjects are presented in Fig. 12, Fig. 13, Fig. 14. In this case, window length calculated using standard SAD approach was even longer, ranging from 490 to 590 s. We can observe very similar loss of detail as in the case of PAMAP2 dataset. Many potentially useful heart rate fluctuations are lost, while only general trend of heart rate is preserved.

Window length based on the 2nd zero point of 3rd gradient of SAD curve ranged from 8 to 16 s. The resulting heart rate signal preserved all the major variations of the original, while removing the noise.

Based on the shape of the heart rate curve, we conclude that using modified SAD approach as proposed in the previous section, preserves characteristic points including shape, absolute value, and relative values.

### Part 2 of validation – longest period

Heart-rate related measures are one of the most commonly used markers in monitoring performance, fitness and fatigue of a person during physical effort [6]. One of the methods of evaluating person's fitness and performance, as well as planning physical training, is based on heart rate zones [7]. Heart rate zones are determined using predefined percentage of maximum heart rate of a person. In automatic analysis of time spend during extended physical effort in certain heart rate zone it is crucial to obtain the whole period of HR above given threshold. For unfiltered HR signal such periods may be erroneously divided into shorter segments due to HR sensor inaccuracy or a single sample below the selected threshold. Application of the proposed method allows to minimize or eliminate such errors.

**Fig. 15:**
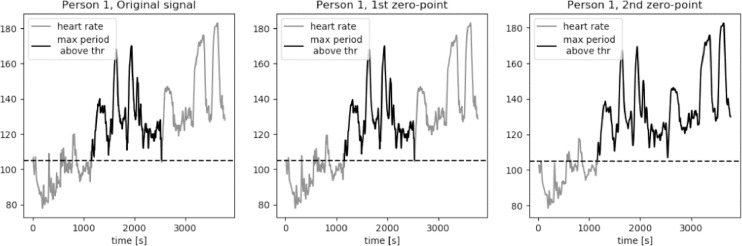
Heart rate: original signal (gray) with highlighted longest period above the threshold (black), signal with moving average filter with window length based on first and second zero-point of third gradient, for Person 1 from PAMAP2 dataset.

**Fig. 16:**
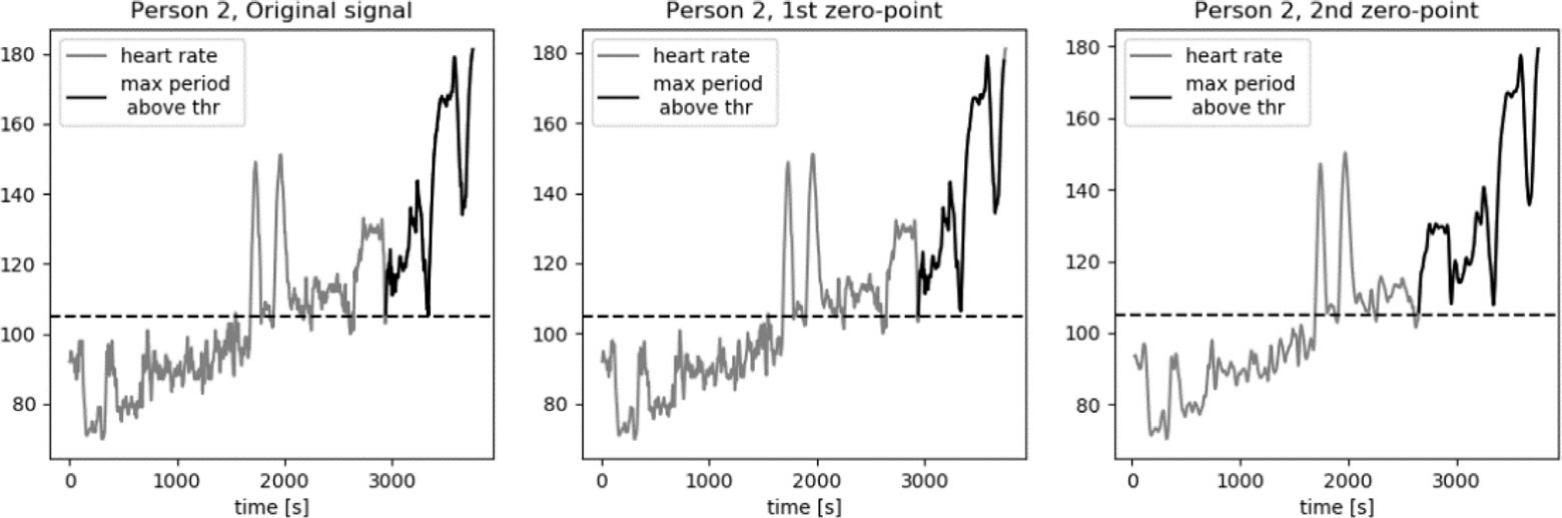
Heart rate: original signal (gray) with highlighted longest period above the threshold (black), signal with moving average filter with window length based on first and second zero-point of third gradient, for Person 2 from PAMAP2 dataset.

**Fig. 17:**
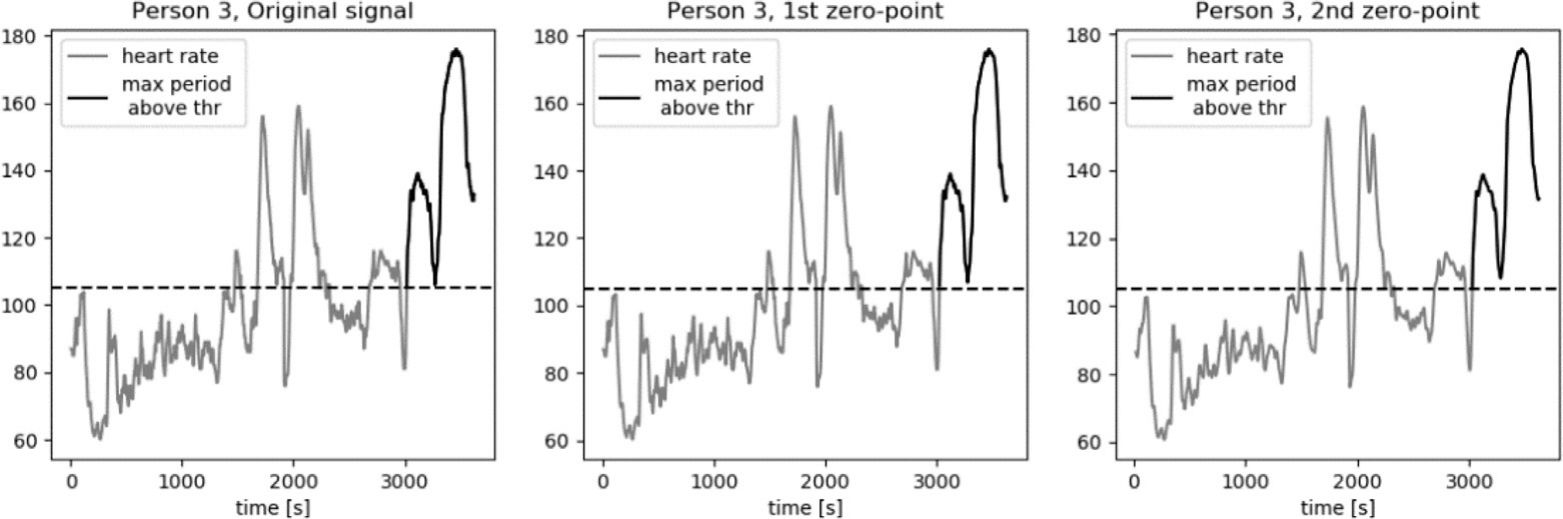
Heart rate: original signal (gray) with highlighted longest period above the threshold (black), signal with moving average filter with window length based on first and second zero-point of third gradient, for Person 3 from PAMAP2 dataset.

**Fig. 18:**
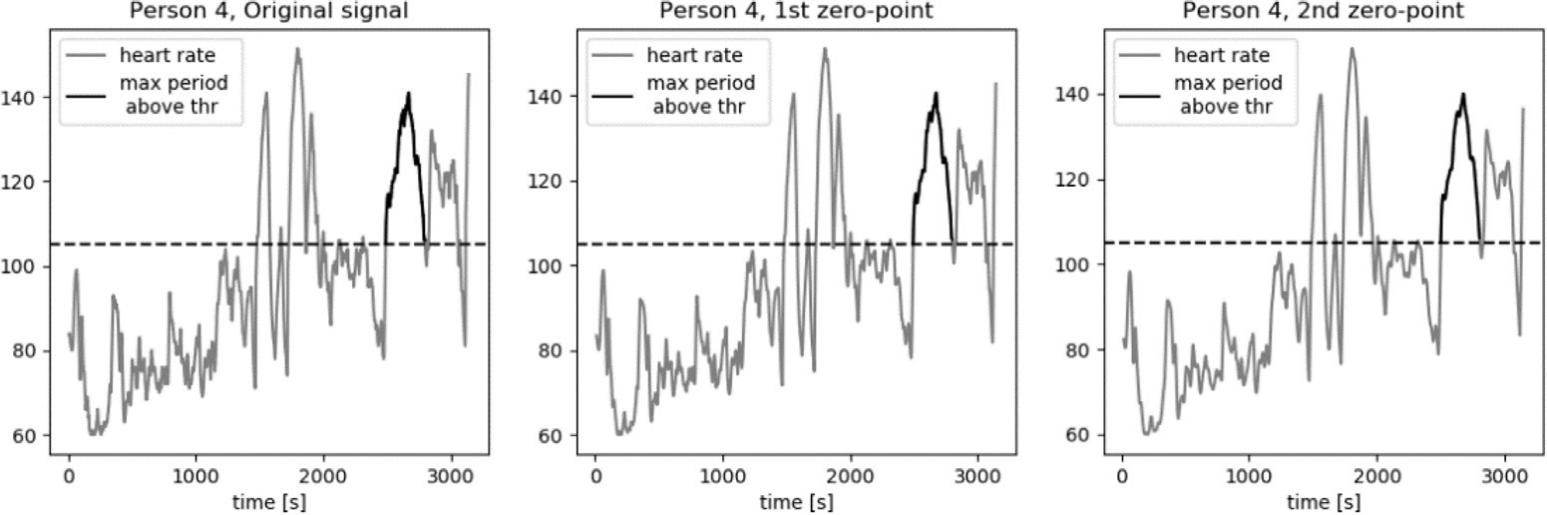
Heart rate: original signal (gray) with highlighted longest period above the threshold (black), signal with moving average filter with window length based on first and second zero-point of third gradient, for Person 4 from PAMAP2 dataset.

**Fig. 19:**
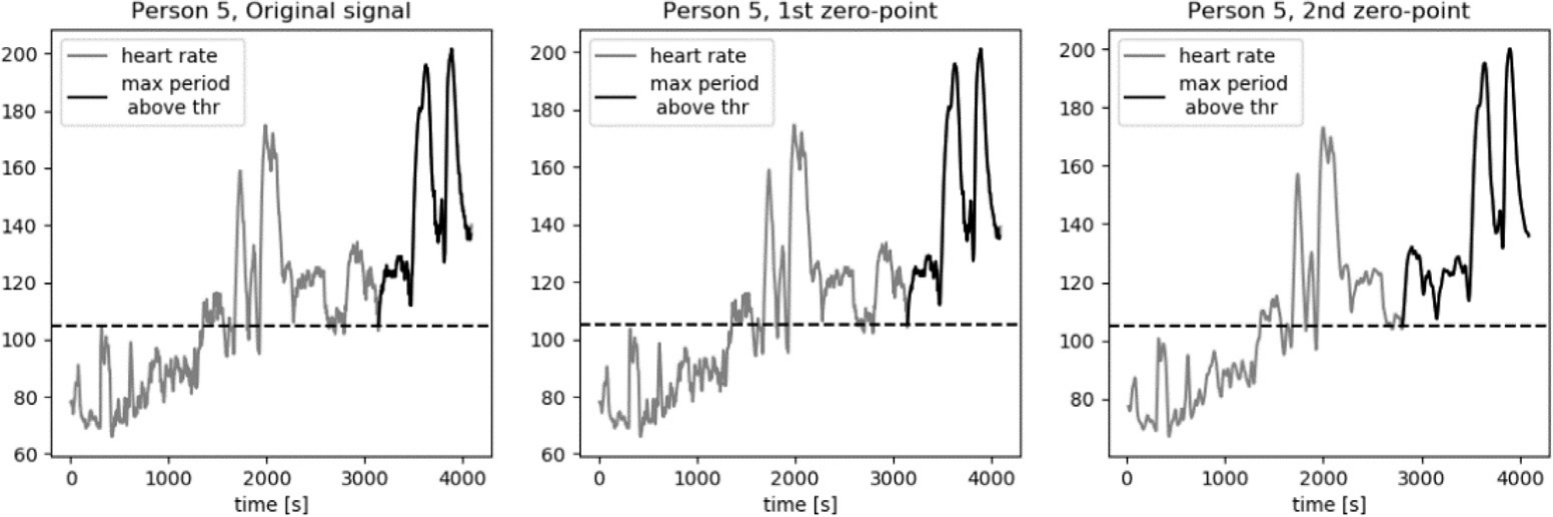
Heart rate: original signal (gray) with highlighted longest period above the threshold (black), signal with moving average filter with window length based on first and second zero-point of third gradient, for Person 5 from PAMAP2 dataset.

For an average middle age person zone 2 of the heart rate, corresponding to the middle exercise intensity, is a typical aerobic target. The bottom value of heart rate in zone 2 is equal to the 60% of a maximum heart rated. We approximate this value to be equal to 105 bpm for average young to middle-aged person. The results of calculations of the longest period of *HR* series above the threshold value *thr* = 105 bpm are presented in Fig. 15, Fig. 16, Fig. 17, Fig. 18, Fig. 19. The first plot in each figure represents the original signal, the second plot shows the results for signal with Moving Average filter with window length based on first zero-point of third gradient, while the last plot presentes the signal with Moving Average filter with window length based on the second zero-point of third gradient. In case of persons 1, 2 and 5 we can see a significant increase of the longest period of the *HR* series above the defined threshold value for the second zero-point of the third gradient. This increase is equal to 90%, 36% and 35% respectively. Including the uncertainty factor UF defined in the previous section, this increase was lower, equal respectively to 8%, 1.7% and 1.8% for persons 1, 2 and 5, as showed in Table 1.

**Table 1:** Longest periods of HR above the threshold value, PAMAP2 dataset.

		Original signal	Filter based on 1st zero-point	Filter based on 2nd zero-point	Filter for 230	Adjusted value
Max. length [*sec*.]	Person 1	1371	1371	2558	2539	1489
	Person 2	812	810	1103	2018	826
	Person 3	606	603	598	797	602
	Person 4	310	308	307	527	308
	Person 5	946	943	1281	2578	963

In case of person 1 we can observe a drop in the heart rate around 2600s., however the heart rate drops slightly below the threshold, by about 1–2 bpm. The behavior of the HR curve suggests that this decrease is insignificant and should not influence the period of heart rate above the threshold of 105 bpm. In case of the original signal and signal filtered using window length corresponding to the first zero-point of the third gradient, this decrease in the heart rate is reducing the period of the HR above the threshold by almost half, from about 43 min to 22 min, what can be crucial during training of a middle aged person.

Cases for person 2 and person 5 are similar, in terms of a slight and short decrease of heart rate, to person 1. In both cases the HR drop below the threshold is by 1–2 bpm and should not be considered in the determination of the value of the longest period above 105 bpm. The increase of this period for persons 2 and 5 for filtered signal with window length equal to the second zero-point is not as large as in case of person one, but still significant, equal to around 5 min.

For persons 3 and 4 the proposed method did not influence the length of the longest period of heart rate above the threshold. Both HR curves show moments of decreased activity, lasting for few seconds, before the considered period in case of person 3, and after the considered period in case of person 4.

It is clearly visible that the longest period above the threshold value calculated for window length equal to 230 is significantly longer in case of persons 1, 2, 3 and 5. For person 1 this value is very similar to the result calculated for the window length corresponding to the second zero-point of the third gradient. However, in case of persons 2 and 5 these values cannot be taken into consideration in analysis of the heart rate in terms of person's performance and fatigue. As we can observe on the first plot of Figs. 16 and 19, there are segments of reduced HR: for person 2 this significant decrease of heart rate is present in segment between 2000s. and 2800s, while in case of person 5 the period of rest occurs between 2600s. and 2900s. In Table 1 we can see that the increase of the period length for person 2 is by 15 min in comparison to the result for the window length based on the second zero-point and by 20 min in comparison to the value obtained for the original signal. In case of person 5 these differenced are even more significant, equal respectively to 21 min and 27 min. Differences at this level of values make filtering of the signal with window length of 230 unreliable.

**Fig. 20:**
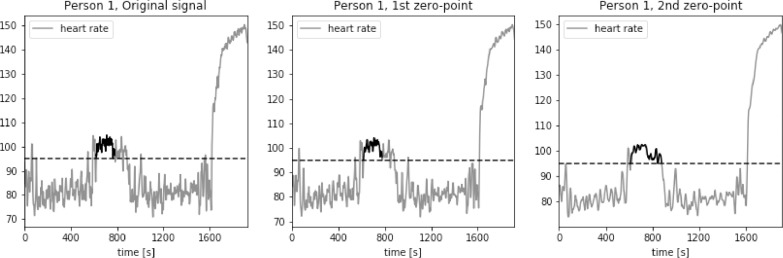
Heart rate: original signal (gray) with highlighted longest period above the threshold (black), signal with moving average filter with window length based on first and second zero-point of third gradient, for Person 1 from Simultaneous Physiological Measurements dataset.

**Fig. 21:**
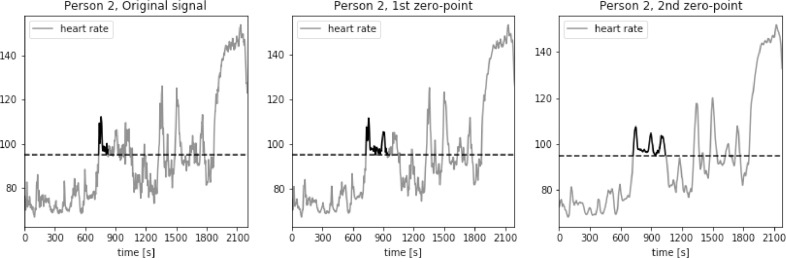
Heart rate: original signal (gray) with highlighted longest period above the threshold (black), signal with moving average filter with window length based on first and second zero-point of third gradient, for Person 2 from simultaneous physiological measurements dataset.

**Fig. 22:**
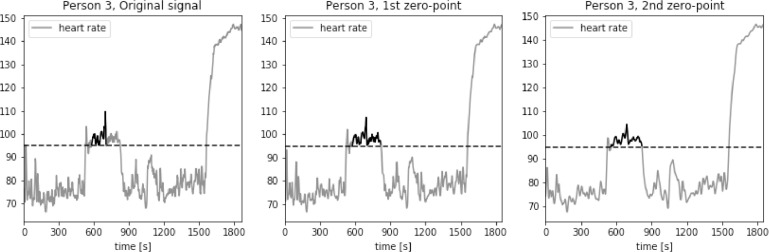
Heart rate: original signal (gray) with highlighted longest period above the threshold (black), signal with moving average filter with window length based on first and second zero-point of third gradient, for Person 3 from simultaneous physiological measurements dataset.

The method was also validated using Physionet dataset “Simultaneous physiological measurements with five devices at different cognitive and physical loads”. The threshold was set to 95 bpm to evaluate the heart rate signal recorded during the period of five-minute walking on the treadmill at a moderate speed. As it can be observed in Fig. 20, Fig. 21, Fig. 22, the signal is very irregular. In case of the original signals the elevated pulse was evaluated to last for less than 3 min in all three cases. For the signals processed using filters based on the second zero-point of the third gradient the resulting longest period increased by 38%, 70% and 53% for persons 1, 2 and 3 respectively. Including the uncertainty factor UF defined in the previous section, this increase was lower, equal respectively to 6%, 68% and 17%, as showed in Table 2.

**Table 2:** Longest periods of HR above the threshold value, simultaneous physiological measurements dataset.

		Original signal	Filter based on 1st zero-point	Filter based on 2nd zero-point	Adjusted value
Max. length [*sec*.]	Person 1	161	161	262	172
	Person 2	93	210	318	217
	Person 3	123	245	262	247

**Fig. 23:**
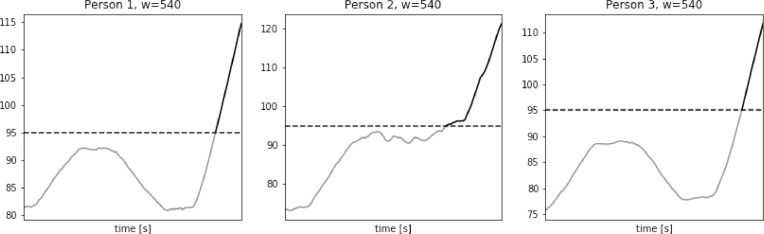
Signal with moving average filter with window length of 540 s, for Persons 1, 2 and 3 from simultaneous physiological measurements dataset.

The longest period above the threshold value calculated for window length equal to 540 for the three analyzed signals is presented in Fig. 23. It can be observed that the signal is significantly smoothed, and its level decreased, what resulted in identification of the region of interest to the incorrect signal part, namely the region when the persons performed uphill walking on the treadmill. Therefore, consideration of the longest period above the threshold, as well as the area under the HR curve and above the threshold in case of the window of such large length is pointless.

### Part 3 of validation – area under the curve

One of the parameters recommended in analysis of serial measurements in medical research is area under the curve. It can be interpreted as a kind of weighted average of the analyzed signal and can be used for finding and evaluating patterns among groups of monitored patients [8].The area between the HR curve and the threshold line was calculated for the original signal, signal with Moving Average filter with window length based on first and second zero-point of third gradient, and length equal to 230. Obtained values are presented in Table 3. We can observe that for the original signal and signals with applied Moving Average filter with window length based on first zero-point of third gradient, are almost the same, with the decreasing tendency for increasing the window length. The reason for it is the fact that the level of the signal decreases with the increase of the length of the window. In case of the filter based on the second zero-point of the third gradient the area under the HR plot and above the threshold value are significantly increased in case of persons 1, 2 and 5, what is a result of the increase of the value of the longest period above the threshold value. The significant difference is visible for the Moving average filter with window of length 230, where the values of the area under the HR curve decreases by 10–20% for persons 3 and 4. Lower amplitudes of the filtered signal can be observed in Fig. 15, Fig. 16, Fig. 17, Fig. 18, Fig. 19, what is also visible in values of the total area under the HR plot as presented in Table 4.

**Table 3:** Area under the HR plot and above the threshold value, PAMAP2 dataset.

		Original signal	Filter based on 1st zero-point	Filter based on 2nd zero-point	Filter for 230	Adjusted value
**Area [heart beats]**	Person 1	34,235	34,233	79,858	76,390	38,772
	Person 2	29,003	28,811	34,178	12,131	29,106
	Person 3	23,674	23,604	23,451	18,692	23,588
	Person 4	6385	6380	6365	8416	6378
	Person 5	40,679	40,611	46,426	66,376	40,952

**Table 4:** Area under the HR plot, PAMAP2 dataset.

		Original signal	Filter based on 1st zero-point (decrease [%])	Filter based on 2nd zero-point (decrease [%])	Filter for 230 (decrease [%])
**Area [heart beats]**	Person 1	467,396	466,812 (0.12%)	465,647 (0.37%)	439,685 (5.93%)
	Person 2	410,011	408,778 (0.30%)	404,328 (1.39%)	356,012 (13.17%)
	Person 3	380,245	379,699 (0.14%)	378,606 (0.43%)	352,732 (7.24%)
	Person 4	302,638	301,955 (0.23%)	301,073 (0.52%)	280,685 (7.25%)
	Person 5	470,828	470,393 (0.09%)	468,573 (0.48%)	444,873 (5.51%)

Obtained results show that proposed method of filtering does not affect significantly the area under the HR curve and this parameter can be used in analysis of the time series. On the other hand, we can see that filtering affects the amplitude of the signal and applying Moving Average filter with long window is not recommended as it significantly affects the area under the curve. This problem might manifest often when measuring heart rate under a load lasting more than several minutes. Due to aforementioned cardiac drift, average heart rate will increase with time even without changes to the exercise load. It will result in the time series of heart rate becoming non-stationary. What will lead to distortion of measurements if a Moving Average filter with long window is applied.

The area under the HR plot and above the threshold value, as well as the total area under the HR plot, were evaluated also for the PhysioNet Simultaneous Physiological Measurements dataset and are presented in Table 5 and Table 6. It is clearly visible that for all the analyzed signals there is an increase of the area under the HR plot and above the threshold value as the length of the longest period above the threshold increases as a result of applying the filter based on the first and second zero-point of the third gradient.

**Table 5:** Area under the HR plot and above the threshold value, simultaneous physiological measurements dataset.

		Original signal	Filter based on 1st zero-point	Filter based on 2nd zero-point	Adjusted value
**Area [heart beats]**	Person 1	900	897	1164	926
	Person 2	548	967	1346	993
	Person 3	482	822	829	823

**Table 6:** Area under the HR plot, simultaneous physiological measurements dataset.

		Original signal	Filter based on 1st zero-point	Filter based on 2nd zero-point	Filter for 540
**Area [heart beats]**	Person 1	179,939	179,353 (0.33%)	178,396 (0.86%)	122,789 (31.76%)
	Person 2	207,506	206,904 (0.29%)	205,481 (0.98%)	151,770 (26.85%)
	Person 3	167,675	167,232 (0.26%)	166,240 (0.86%)	113,015 (32.59%)

The values of the total area under the HR plot have slightly decreasing tendency as the size of the window increases, however this decrease is not very significant for the filters based on the first and second zero-point of the third gradient. It is much greater in case of the window size of 540 and is equal to 20–30%, as it can be seen in Table 6.

## Concluding remarks

Sum of absolute differences is an established approach to selecting window length for a moving average filter. The underlying properties of a process generating the data might, however, render results of such approach detrimental to further analysis for some signals. Biomedical signals, such as heart rate, are a good example, since underlying physiology is often well understood. As we demonstrated regular SAD approach results in long windows that undesirably remove physiological information from the data.

The method outlined in this paper provides a formalized alternative to the regular sum of absolute difference approach. It extends it with calculation of zero-points of third gradient, sometimes called jerk, to detect finer variations in the shape of SAD curve.

We demonstrated validity of this approach on the PAMAP2 Physical Activity Monitoring Data Set, an open dataset from the UCI Machine Learning Repository, as well as on the PysioNet Simultaneous Physiological Measurements dataset. It showed that first zero-point usually falls at around 8 and 5 second window length respectively, while second zero-point usually falls between 16–24 and 8–16 s respectively. The value for the first zero-point can remove simple measurement errors when data are recorded once every few seconds. The value for the second zero-point corresponds well with what is known about physiological response of heart to changing load [9].

## Declaration of Competing Interest

The authors declare that they have no known competing financial interests or personal relationships that could have appeared to influence the work reported in this paper.
